# Perioperative management of cesarean section in pregnant women complicated by central core disease: A case report and literature review

**DOI:** 10.1097/MD.0000000000042089

**Published:** 2025-04-04

**Authors:** Sipei Cheng, Rou Yu

**Affiliations:** a Department of Anesthesiology, West China Second University Hospital, Sichuan University, Chengdu, China; b Key Laboratory of Birth Defects and Related Diseases of Women and Children, Ministry of Education, Sichuan University, Chengdu, China.

**Keywords:** case report, central core disease, pregnancy, RYR1 gene

## Abstract

**Rationale:**

The central core disease (CCD) is a relatively uncommon yet frequently encountered condition characterized by slow or nonprogressive weakness primarily affecting the proximal limbs, predominantly observed during infancy or childhood. Pregnancy combined with CCD is an exceedingly rare occurrence, which needs a multidisciplinary approach for perinatal management. However, there remains considerable debate regarding the optimal timing and methodology for pregnancy termination as well as the utilization of anesthesia or sedatives.

**Patient concerns:**

We present a case of a patient complicated by CCD, in which we also identified a family carrying a missense mutation of RYR1 (NM_000540) c.13910 C>T (exon 95), p.T4637I (heterozygous). The patient uneventfully delivered a female neonate via cesarean section under continuous epidural anesthesia.

**Diagnoses:**

The patient, a 27-year-old pregnant woman with complications of CCD and a missense mutation of RYR1, expressed the desire to terminate her pregnancy.

**Intervention:**

Multiple protocols of anesthesia management were developed based on the patient’s specific condition and surgical requirements, which included scheduled surgery under neuraxial anesthesia, as well as preparation of general anesthetic drugs, dantrolene sodium, airway devices, and specialized anesthesia machines for emergencies such as cord prolapse or fetal bradycardia.

**Outcomes:**

The cesarean section went smoothly with continuous epidural anesthesia at the L3–4 and T12–L1 intervertebral space with the catheter inserted upwards at T12–L1, and downwards at L3–4. The patient and her baby were discharged after 4 days without any complications related to anesthesia.

**Lessons:**

The identification of a CCD family in our case not only contributes to a deeper understanding of anesthesia methods in CCD pregnant women but also enriches the variation database of the RYR1 gene, which is essential for conducting long-term follow-up studies.

## 1. Introduction

Central core disease (CCD) (On-line Mendelian Inheritance in Man, OMIM #11700) is a rare genetic myopathy with an estimated prevalence of approximately 6 per 100,000 live births. It is characterized by slow or nonprogressive proximal limb skeletal muscle weakness and hypotonia, muscular atrophy,^[1]^ and skeletal deformities such as hip dislocation, scoliosis, and foot deformities including talipes equinovarus and flat foot, etc.^[2,3]^ The major gene mutation encoding the skeletal muscle ryanodine receptor (RYR1 on chromosome 19q13.1) is implicated in this disease,^[4]^ which also results in susceptibility to malignant hyperthermia (MH), and reports an increased risk of MH in patients with CCD.^[5,6]^ It is extremely rare for a pregnancy to be combined with CCD. The weakened muscle strength, pelvic narrowing, and scoliosis will make transvaginal delivery impossible, thus requiring cesarean section to terminate pregnancy in most patients.^[7]^ The use of anesthesia or sedatives has also been widely discussed. The skeletal deformities, such as hip dislocation and scoliosis, will lead to difficulties in regional anesthesia. General anesthesia poses challenges with difficult airway management and MH. In addition, the choice of anesthetic techniques and medication effects on the fetus must also be considered in emergencies.

## 2. Case presentation

**Figure 1.**
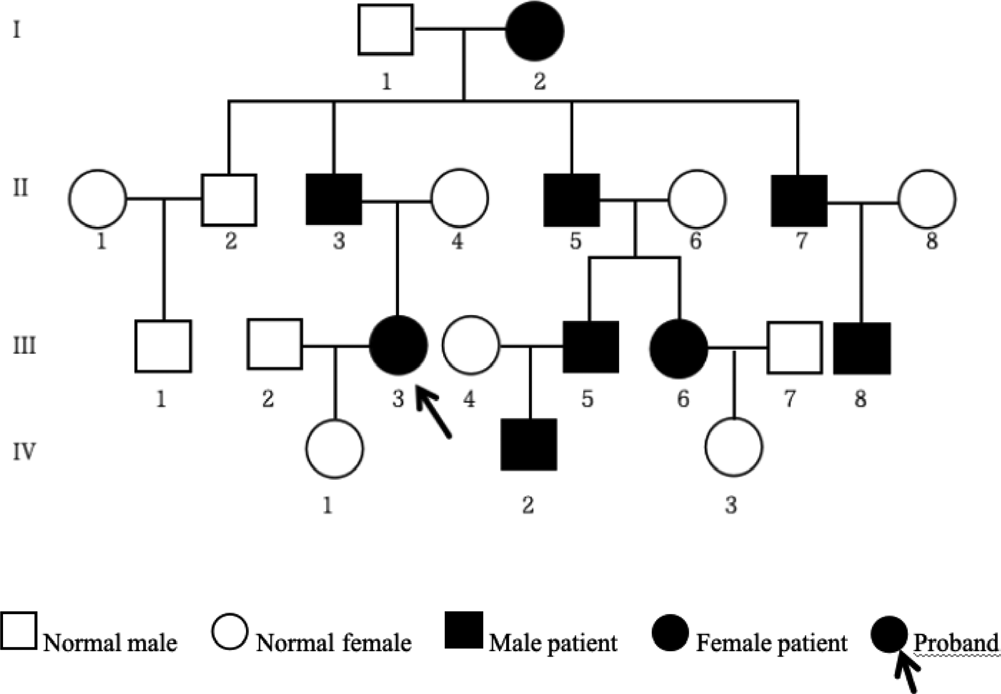
The family’s genetic genealogy.

A 27-year-old woman at 37 weeks’ gestation with an RYR1 gene abnormality was scheduled for a cesarean section. Before that, the patient underwent advanced in vitro fertilization and embryo transfer techniques of the third generation. She was admitted to the genetics department at the age of 5 due to “difficulties in ascending stairs,” despite the absence of a clear diagnosis. However, her muscle strength continued to decline in both lower limbs. Subsequent gene analysis performed on the entire family confirmed the presence of a missense mutation in RYR1 (NM_000540) c.13910 C>T (exon 95), p.T4637I (heterozygous), which is associated with an increased predisposition to CCD, leading to varying degrees of limb muscle weakness among the patient, her father and his younger brothers, as well as her cousins and her younger cousin’s daughter. Additionally, an investigation into their familial background revealed comparable symptoms exhibited by their late grandmother, who had been strongly suspected of having CCD before succumbing to a cerebral hemorrhage. The genetic genealogy of the family is illustrated in Figure 1. The patient reported herein has previously undergone 2 anesthesia and surgical procedures. In childhood, she underwent the removal of a left kidney stone under continuous epidural anesthesia, her bilateral Achilles tendon length under total intravenous anesthesia, and no exposure to muscle relaxants and inhalants.

**Figure 2.**
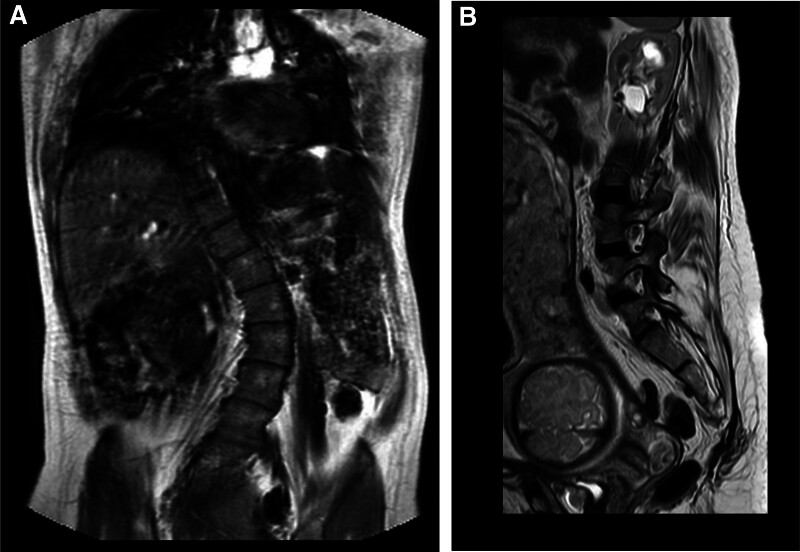
Thoracolumbar MRI. The thoracolumbar vertebrae exhibited an “S” shaped lateral curvature. The spinal cord within the spinal canal appeared smooth, with the conus medullaris positioned at the level of the L1 vertebra, and no space-occupying lesions were detected. There was a slight dilation in the left kidney along with a renal cyst. MRI = magnetic resonance imaging.

The patient presents with a height of 146 cm and a weight of 52 kg. Physical examination revealed a grade 4 strength level in the bilateral lower limbs, accompanied by severe scoliosis and lameness. However, there were no objective weaknesses observed in the facial, pharyngeal, or masseter muscles. Furthermore, she did not exhibit symptoms of chest tightness, hypoxia, or breathlessness. During this pregnancy, the patient experienced no complications other than being diagnosed with gestational diabetes mellitus at 23 weeks of gestation. Upon admission, a spinal magnetic resonance imaging (Fig. 2) was performed on the patient, results indicated no occupying lesions within the vertebrae. The conus medullaris was located at the L1 vertebra level and the Cobb angle measured 25°. Both electrocardiogram and echocardiogram results were normal. However, an ultrasound of the lower limb vein revealed sluggish blood flow to the great saphenous vein. Standard laboratory tests indicated a normal creatine kinase level of 50 U/L. Following multidisciplinary consultations involving obstetrics, anesthesia, and neurology among others, an elective cesarean section was scheduled for pregnancy termination based on both clinical considerations and patient preference.

We have developed a comprehensive range of anesthesia management programs tailored to the specific condition and surgical requirements of the patients, including intrathecal anesthesia for elective cesarean section, as well as the preparation of general anesthetic drugs, dantrolene sodium, airway devices, and specialized anesthesia machines for emergencies such as cord prolapse or fetal bradycardia. Initial vital signs were recorded as follows: noninvasive blood pressure of 118/77 mm Hg, heart rate of 80 beats per minute, and room air SpO2 of 95%. The patient was positioned in left lateral decubitus and we could touch the T12–L1 and L2–4 intervertebral spaces. Due to the difficulties of inserting the spinal needle at L3–4, we chose continuous epidural anesthesia at both T12–L1 and L2–3 levels. Following a test dose of 3 mL (1.5% lidocaine and 1:200,000 epinephrine), an epidural administration of 21 mL of 3% chloroprocaine (13 mL in the upper tube and 8 mL in the lower tube) was given. Once the sensory blockade reached T5, the cesarean section proceeded smoothly with the delivery of the fetus within 2 minutes. The Apgar scores at both 1 minute and 5 minutes after birth were recorded as perfect tens. The surgical procedure lasted for 38 minutes, and the effect of anesthesia was satisfactory. Since the patient might need a subcutaneous injection of heparin to prevent thrombosis postoperatively, so we removed the epidural catheter. Patient-controlled intravenous analgesia and transversus abdominis plane block techniques (0.25% ropivacaine 20 mL with 2.5 mg dexamethasone per side) were employed for postoperative pain management. The patient remained stable throughout and was discharged 4 days after surgery.

## 3. Discussion

The term “congenital myopathies” refers to a spectrum of muscle disorders inherited in an autosomal dominant or recessive manner. These disorders can vary significantly in presentation—from asymptomatic to progressive conditions affecting multiple organ systems. Notable examples include nemaline myopathy, central core disease, multi-mini core disease, centronuclear/myotubular myopathy, and congenital fiber-type disproportion.^[8]^ During pregnancy in women with CCD, the affected fetuses, particularly those inheriting the same genetic abnormality, may experience polyhydramnios, premature birth, impaired motor function, or floppy infant syndrome. Maternal progesterone levels increase during pregnancy, which can temporarily exacerbate muscle weakness. Furthermore, scoliosis can further aggravate restrictive respiratory insufficiency^[9]^; therefore it is crucial to regularly monitor oxygen saturation and respiratory intensity in pregnant women with severe myopathy.

The diagnosis of CCD primarily relies on muscle biopsy to identify the presence of the centrally located nucleus and indicative clinical manifestations. However, in clinical practice, performing muscle biopsies can pose challenges due to the relatively low compliance among pediatric patients and their families. The structure of muscle cells and nerve tissue exhibits high heterogeneity, which is significantly influenced by genetic factors during development. Therefore, the vast majority of diagnoses are primarily based on genetic testing and clinical presentation. The RYR1 gene (19q13.1, OMIM #180901) has currently been identified as a prominent pathogenic gene in CCD and is strongly associated with MH. Located on chromosome 19q13.1, RYR1 encodes a tetrameric calcium channel protein that plays a pivotal role in excitation–contraction coupling and calcium homeostasis within skeletal muscle. Mutations associated with CCD primarily occur within the C-terminal region of the gene (exons 85–103), exhibiting a higher prevalence within specific families rather than being widely distributed across multiple families (<20%).^[10]^ Individuals affected by this condition should use second-generation genetic sequencing techniques for early genetic testing, genetic counseling, and prenatal diagnosis to disrupt the chain of inheritance and alleviate burdens on both families and society. The p.T4637I variant identified in our study may represent a novel genetic mutation, contributing valuable information to the RYR1 gene mutation database.

To date, research on successful pregnancies among patients with CCD remains relatively limited, and some cases may have gone undiagnosed or unreported. For women diagnosed with CCD, there is ongoing debate regarding the optimal timing and method for pregnancy termination, as well as the use of anesthesia or sedatives during the procedure. Due to weakened muscle strength, pelvic narrowing, and scoliosis, transvaginal delivery is not feasible for terminating the pregnancy in these cases; therefore, cesarean section becomes necessary.^[7]^ A comprehensive review published in 2019 summarized 29 pregnancies in 21 patients with morphologically defined CM, including 6 patients with CCD. Among these, there were 3 elective cesarean sections performed under different anesthesia methods: one under general anesthesia due to severe scoliosis, one using combined spinal cord and epidural anesthesia, and 3 cases where the type of anesthesia was unspecified.^[11]^ In another case reported in Japan in 2020, a cesarean section was performed under total intravenous anesthesia for a patient with a history of scoliosis.^[12]^

In general, regional anesthesia is preferable to general anesthesia in patients with CCD.^[13]^ The utilization of neuraxial anesthesia can effectively mitigate the risks associated with intubation failure and extubation difficulty during general anesthesia, while also minimizing adverse effects on the circulatory system and ensuring sustained postoperative pain relief. However, in patients with severe spinal deformities or surgically corrected scoliosis, achieving a successful epidural blockade presents challenges due to the intricate task of accurately locating the puncture site. Moreover, horizontal rotation and lateral angle of the patient’s vertebrae pose exceptional difficulties in adjusting needle insertion angles. Ultrasound-guided spinal anesthesia is a valuable modality for obtaining crucial information that may help address technical challenges encountered in patients with atypical spinal anatomy.^[14]^ Alterations in the ligamentous structures surrounding the spine and the presence of scar tissue impede drug diffusion, thereby impacting the efficacy of anesthesia. General anesthesia becomes inevitable when regional anesthesia proves unsuccessful or during emergent situations such as umbilical cord prolapse or fetal bradycardia.

Patients with CCD may exhibit facial dysmorphisms, such as retrognathism and a high-arched palate, which can pose challenges in airway management. Moreover, CCD has been identified as a potential driver of MH, particularly in patients with elevated levels of serum creatine kinase. All these factors contribute to an increased risk during general anesthesia procedures. Before administering anesthesia, it is crucial to get ready a difficult airway cart, which should be equipped with a range of tracheal tubes in various sizes, both single-lumen and double-lumen laryngeal masks, video laryngoscopy equipment, fiberoptic bronchoscopes, and lighted stylets, as demonstrated in our case. Before surgery, replace the anesthesia breathing circuit and sodium hydroxide canister, remove the evaporator, and rinse the anesthesia machine with 10 L/min of air for 12 hours.^[15]^ The essential components for preparation include dantroline sodium, ice packs, saline solution, and continuous intraoperative monitoring of core body temperature. Triggering agents like inhaled anesthetics (e.g., isoflurane, sevoflurane, and halothane) and succinylcholine should also be avoided.^[16]^ Residual neuromuscular blockade can lead to postoperative respiratory failure; therefore, the implementation of quantitative neuromuscular monitoring is necessary for prevention. Typically, the adductor pollicis muscle is the standard site for assessing neuromuscular blockade. The craniofacial and cervical muscles play a crucial role in maintaining airway patency, and patients with CCD may have potential muscle weakness. Thus, some studies have also employed neuromuscular monitoring at the masseter in addition to the adductor pollicis.^[15]^

In conclusion, perioperative anesthetic management strategies for patients with CCD should be personalized based on the specific circumstances of both the mother and baby. Both general anesthesia and neuraxial anesthesia are viable approaches that can be considered. The identification of a family with CCD in our case not only contributes to a deeper understanding of anesthesia methods applicable to those patients but also enhances the variation database of the RYR1 gene, which is crucial for conducting long-term follow-up studies.

## Author contributions

**Conceptualization:** Sipei Cheng.

**Data curation:** Rou Yu.

**Writing – original draft:** Sipei Cheng.

**Writing – review & editing:** Rou Yu.
